# Negative illness perceptions are related to poorer health‐related quality of life among thyroid cancer survivors: Results from the PROFILES registry

**DOI:** 10.1002/hed.26290

**Published:** 2020-06-03

**Authors:** Dounya Schoormans, Laura Wijnberg, Harm Haak, Olga Husson, Floortje Mols

**Affiliations:** ^1^ CoRPS—Center of Research on Psychological and Somatic disorders, Department of Medical and Clinical Psychology Tilburg University Tilburg The Netherlands; ^2^ Department of Internal Medicine Maxima Medical Centre Eindhoven The Netherlands; ^3^ Institute of Cancer Research and Royal Marsden NHS Foundation Trust London UK; ^4^ Department of Psychosocial Research, Division of Psychosocial Research & Epidemiology The Netherlands Cancer Institute Amsterdam The Netherlands; ^5^ Department of Research Netherlands Comprehensive Cancer Organisation (IKNL) Utrecht The Netherlands

**Keywords:** cancer survivors, differentiated thyroid cancer, health‐related quality of life, illness perceptions, PROFILES

## Abstract

**Background:**

Differentiated thyroid cancer (DTC) reports a poorer health‐related quality of life (HRQoL) than a norm population. Patients' illness perceptions are modifiable and known associates of HRQoL in other cancers. The aim was to examine the relationship between illness perceptions and HRQoL among DTC survivors.

**Methods:**

DTC survivors registered in the Netherlands Cancer Registry diagnosed between 1990 and 2008, received a survey on illness perceptions (Brief‐Illness Perception Questionnaire; B‐IPQ) and HRQoL (European Organisation for Research and Treatment of Cancer, Quality of Life Questionnaire‐Core 30; EORTC QLQ‐C30). Multiple regression analyses were conducted investigating the relation between illness perceptions and HRQoL, while controlling for sociodemographic and clinical characteristics.

**Results:**

Two hundred and eighty‐four DTC survivors were included. DTC survivors who believed their illness had many negative consequences; who perceived their illness as controllable by treatment; who had strong beliefs symptoms could be attributed to their illness; and who had strong beliefs their illness causes negative emotions, reported a poorer HRQoL.

**Conclusions:**

Stronger negative illness perceptions are related to a poorer HRQoL among DTC survivors.

## 1 INTRODUCTION

Thyroid cancer is the most frequent malignancy of the endocrine system, with the differentiated thyroid cancers (DTC) papillary and follicular accounting for the majority of new cases.^1^ The incidence of DTC is 2.5 times higher in women than in men,^2^ and the five‐year survival rate is 98.1%.^3^ The most common treatment of DTC is a total thyroidectomy typically followed by radioactive iodine treatment which aims to target remaining malignant thyroid cells.^4^ Removal of the thyroid gland results in thyroid replacement therapy throughout one's entire life.^5^

Although DTC has a high survival rate and some studies report HRQoL similar to that of non‐cancer populations,^6^, ^7^ recent findings show a poorer HRQoL among DTC survivors compared to other cancers (ie, breast, prostate, uterine, cervical, colorectal, lung, and non‐Hodgkin lymphoma).^8^ A previous study of our group examined HRQoL among long‐term DTC survivors and found that DTC survivors reported significantly worse levels of physical and psychological functioning, as well as a significantly higher number of symptoms compared to a normative population.^9^, ^10^

Identifying modifiable associates through which HRQoL of DTC survivors can be improved is an important area of research. How patients perceive their illness is a known determinant of HRQoL in various patient groups including cancer.^11^, ^12^, ^13^ These illness perceptions are hypothesized to influence illness adaptation and outcomes within the structure of the common‐sense self‐regulation model (SRM) of illness.^14^ According to the SRM model, when individuals are faced with an illness, they form cognitive representations (beliefs about the illness) and emotional reactions which together affect both physical and psychosocial outcomes, principally through coping responses.^14^

Previous studies among various cancer populations not including DTC, demonstrated that cancer survivors with optimistic illness perceptions regarding their prognosis had better health outcomes compared to those with pessimistic and realistic Illness perceptions.^13^ Among various malignancies (breast, prostate, and colorectal cancer) negative illness perceptions were related to poorer HRQoL.^15^, ^16^ Furthermore, previous studies provide promising evidence for the effectiveness of interventions aimed at helping these individuals acquiring more adaptive illness perceptions, thereby improving their HRQoL.^17^, ^18^, ^19^ A previous study examining illness perceptions among DTC survivors showed that illness perceptions were often unrelated to the clinical objective severity of the disease, and suggested that understanding and referring to illness perceptions of patients with DTC is critical as they could strongly impact their perceived health and HRQoL.^20^ Previous studies of our group have shown that illness perceptions are unrelated to medication adherence^21^ and function as a mediator for the relation between information provision and perceived distress.^22^ Furthermore, we previously concluded that the impact of DTC on HRQoL is greater among younger than older survivors.^10^ However, to our knowledge the relation between illness perceptions and HRQoL has never been examined among DTC survivors. The current study is therefore the first to examine the relation between illness perceptions and HRQoL among a sample of mid to long‐term (2‐20 years after diagnosis) DTC survivors. Knowledge on which illness perceptions are associated with a poor HRQoL is helpful as illness perceptions can be modified by psychological interventions such as cognitive‐behavioral therapy, which may in turn have beneficial effects on DTC survivors' HRQoL.

## 2 MATERIALS AND METHODS

### 2.1 Setting and population

This study is a population‐based survey among DTC survivors who are registered within the Netherlands Cancer Registry (NCR) of the Netherlands Comprehensive Cancer Organisation. The NCR assembles data of all individuals recently diagnosed with cancer in the Netherlands. Every single NCR‐registered individual diagnosed with DTC between 1990 and 2008 in the Southern part of the Netherlands (2.3 million inhabitants) was qualified to participate in the study (N = 568). Exclusion criteria were cognitive impairment or being too ill at the time of the study in order to participate (N = 31), having unverifiable addresses (N = 90), or having deceased before the study began (according to the NCR, hospital records, and the Central Bureau for Genealogy, N = 6). Additionally, one hospital refused to take part in this study (N = 86). The remaining DTC survivors (N = 355) were invited to participate in the study.

### 2.2 Data collection

In November 2010, data was collected via the Patient Reported Outcomes Following Initial treatment and Long term Evaluation of Survivorship (PROFILES) registry. PROFILES is a registry which facilitates studies examining the physical and psychosocial impact of cancer as well as its treatment.^23^ PROFILES includes an extensive web‐based component which is combined with the clinical data from the NCR. Data registered in the NCR has been extracted from medical records by trained registry personnel. Internal validation studies are performed evaluating the quality by randomly checking completeness and accuracy of the registry personnel extracting information from the medical records.^24^ The quality of this data is high because of thorough training of the registrars and computerized consistency checks at regional and national level. Completeness is estimated to be at least 95%.^24^ PROFILES registry data can be freely used for noncommercial scientific research, subject to privacy and confidentiality regulations (www.profilesregistry.nl). Details on the data collection have been published previously.^9^

Participants who were qualified for the study were informed by a letter from their (ex‐)treating physician. Within this letter, the goal of the study was explained and survivors were invited to participate. Additionally, a link was included to a secure website along with a login name, and a password through which interested survivors could grant informed consent and complete the online questionnaires. A paper‐and‐pencil version of both the informed consent form and the questionnaire was available for survivors who preferred that over a digital version. Furthermore, nonrespondents were sent a reminder letter as well as a paper‐and‐pencil version of the questionnaire within 2 months after they had initially been invited to do so. Survivors were guaranteed that there would be no consequences regarding their treatment or follow‐up care if they eventually decided to not participate. This study was reviewed by the Institutional Review Board and was deemed nonhuman subjects research.

### 2.3 Measurement

#### 2.3.1 Sociodemographic and clinical characteristics

The NCR registers sociodemographic (age and gender) and clinical characteristics (time since diagnosis, tumor type, cancer stage via TNM classification, and primary treatment). A questionnaire was used to assess the sociodemographic variables; marital status, educational level, and work status. Comorbidity at the time of the study was assessed with the adapted Self‐administered Comorbidity Questionnaire and included stroke, heart disease, high blood pressure, lung disease, diabetes, ulcer or stomach disease, kidney disease, anemia or other blood disease, depression, osteoarthritis, back pain, and rheumatoid arthritis.^25^

#### 2.3.2 Illness perceptions

The Brief Illness Perception Questionnaire (B‐IPQ) was used to assess the cognitive and emotional perceptions and illness understanding.^26^ The single items are rated on a Likert‐scale from 0 to 10, with higher scores indicating negative illness perceptions. The first five items assess *cognitive perceptions*: (a) How much does your illness affect your life (consequences); (b) How long do you think your illness will continue (timeline); (c) How much control do you feel you have over your illness (personal control); (d) How much do you think your treatment can help your illness (treatment control); and (e) How much do you experience symptoms from your illness (identity). Two items evaluate the *emotional perceptions*: (f) How concerned are you about your illness (concern); and, (g) How much does your illness affect you emotionally (emotional representation). Finally, one item evaluates the degree of understanding of the disease: (h) How well do you understand your illness (*disease understanding*). The B‐IPQ is of good psychometric quality.^26^

#### 2.3.3 Health‐related quality of life

HRQoL was measured by the European Organization for Research and Treatment of Cancer Quality of life Questionnaire‐Core 30‐questions (EORTC QLQ‐C30).^27^ This 30‐item questionnaire comprises a global QoL scale; five functioning scales (physical, role, emotional, cognitive, and social); three symptoms scales (fatigue, nausea and vomiting, and pain), and six single items (dyspnoea, insomnia, appetite, constipation, diarrhea, and financial impact). After linear transformation, all scales and single item measures ranged from 0 to 100 where high scores on the global QoL and functional scales are indicative of greater functioning, whereas a high score on the symptom scales means there are more complaints.

### 2.4 Statistical analyses

Sociodemographic and clinical characteristics were calculated using descriptive statistics. Multiple regression analyses were conducted in order to examine the extent to which the eight illness perceptions (consequences, timeline, personal control, treatment control, identity, concern, emotional representation, and disease understanding) were related to the different HRQoL scales. Multiple regression analyses were conducted for each HRQoL scale (the five functioning scales, the three symptom scales, the global quality of life scale and the six single items) separately. In all analyses we controlled for sociodemographic (age, sex, marital status, educational level, and current occupation) and clinical characteristics (time since diagnosis, type of DTC, primary treatment, and comorbidities), as these factors are known to be related to illness perceptions and/or HRQoL. Clinically meaningful effects for the EORTC QLQ‐C30 scales were determined using EORTC guidelines, which were developed by a systematic review of 152 cancer‐specific articles, expert opinions, and meta‐analyses.^28^ These EORTC guidelines classifies four effects: Trivial, circumstances unlikely to have any clinical relevance or where there was no difference; Small, subtle but nevertheless clinically relevant; Medium, likely to be clinically relevant but to a lesser extent; Large, one representing unequivocal clinical relevance.^28^ As we performed multiple tests, we chose a significant level of 1% as our hypotheses were theory driven and this is the first study relating illness perceptions to HRQoL among DTC survivors. Analyses were performed using IBM SPSS version 24.0 (Statistical Package for Social Sciences, Chicago, Illinois).

## 3 RESULTS

### 3.1 Sociodemographic and clinical characteristics

Of the total 334 DTC survivors, 285 survivors successfully completed the questionnaire (response rate = 85%). As one participant was excluded due to traceability, a total of 284 DTC survivors were included. There were no differences on sociodemographic or clinical characteristics, when comparing respondents to nonrespondents.^22^ The majority of participants were female (76.4%), and the most common type of DTC was papillary (76.1%). A full description of sociodemographic and clinical characteristics is provided in Table 1.

**TABLE 1:** Sociodemographic and clinical characteristics of DTC survivors

	Total population (N = 284)
*Sociodemographics*	
Sex (male)	67 (23.6)
Mean age at time of survey	55.98 (14.12)
Marital status	
Married/cohabiting	224 (78.9)
Divorced/widowed/never married	60 (21.1)
Educational level^a^	
Lower education	30 (10.6)
Medium education	177 (62.3)
Higher education	80 (26.2)
Unknown	2 (0.7)
Work status^b^	
Employed	147 (51.8)
Unemployed	132 (46.5)
Unknown	5 (1.8)
*Clinical characteristics*	
Tumor type	
Papillary	216 (76.1)
Follicular	68 (23.9)
Time since diagnosis in years	
2‐5	73 (25.7)
5‐10	79 (27.8)
>10	132 (46.5)
Cancer stage	
I	168 (57.2)
II	51 (18.0)
III	43 (6.0)
IV	17 (6.0)
Unknown	5 (1.8)
Primary treatment	
Surgery alone	67 (23.6)
Surgery + ablation	207 (72.9)
Surgery + radiotherapy	8 (2.8)
Other	2 (0.7)
Number of comorbid conditions	
None	78 (27.5)
1	83 (29.2)
≥2	123 (43.3)

*Note:* Part of this table has been previously published.^22^

a Education level: low (primary school or no education), medium (lower general secondary education or vocational training), and high (high vocational training or university level).

b Work status: unemployed also includes students, retired individuals, housewives, and so forth.

### 3.2 Illness perceptions and health‐related quality of life

Table 2 provides the mean scores and standard deviations for illness perceptions and HRQoL. DTC survivors hold the strongest negative perception for the item *timeline* (mean of 6.74, SD = 3.79), with higher scores indicate that DTC survivors believe their illness will continue for a long time. The least negative perceptions were for *treatment control* (mean = 3.38, SD = 2.63), with low scores indicating that DTC survivors believe treatment is very helpful, and *emotional perceptions* (mean of 3.38, SD = 2.56), where low scores indicate that DTC survivors are to some extent emotionally affected by their illness.

**TABLE 2:** Mean scores for illness perceptions and health‐related quality of life

	Mean scores	SD
*Illness perceptions*		
Consequences	3.87	2.51
Timeline	6.74	3.79
Personal control	5.41	3.14
Treatment control	3.38	2.63
Identity	4.07	2.79
Concern	3.79	2.55
Emotional representation	3.38	2.56
Illness understanding	3.51	2.60
*Health‐related quality of life*		
Global QoL	75.95	20.19
Functioning		
Role functioning	82.43	26.47
Physical functioning	83.50	19.01
Cognitive functioning	82.18	22.28
Social functioning	85.43	24.62
Emotional functioning	83.64	19.93
Symptoms		
Fatigue	28.01	25.02
Nausea	3.82	10.64
Pain	16.37	24.80
Dyspnoea	13.50	22.01
Insomnia	21.82	29.22
Appetite	5.83	17.67
Constipation	9.40	19.63
Diarrhea	6.33	16.60
Financial impact	9.35	21.39

*Note:* Scores for illness perceptions range on a scale from 0 to 10, with higher scores indicating stronger negative perceptions. HRQoL‐scales and single item measures ranged from 0 to 100 where high scores on global QoL and the functional scales are indicative of greater functioning, whereas a high score on the symptom scales means there are more complaints.

Abbreviation: QoL, quality of life

With respect to HRQoL, survivors reported the highest score on social functioning (mean = 85.43, SD = 24.62), and lowest on global QoL (mean = 75.95, SD = 20.19). As for the symptoms, most often reported were fatigue (mean = 28.10, SD = 25.02), and insomnia (mean 21.82, SD = 29.22).

### 3.3 The relationship between illness perceptions and health‐related quality of life

DTC survivors with stronger beliefs their illness affected their lives (*consequences*) reported poorer global QoL (*B* = −1.95), physical (*B* = −1.97), role (*B* = −3.03), and social (*B* = −2.31) functioning, whereas more fatigue (*B* = 3.13), and pain (*B* = 2.59), Table 3. A stronger belief the illness could be managed by treatment (*treatment control*) was significantly related to lower global HRQoL (*B* = −1.67), and physical functioning (*B* = ‑1.29). DTC survivors who reported experiencing more symptoms as a result of their illness (*identity*), reported poorer social functioning (*B* = −1.83), more fatigue (*B* = 2.15), insomnia (*B* = 2.50), constipation (*B* = 1.55), and experienced a negative financial impact of their illness (*B* = 1.95). Survivors who were affected by their illness (*emotional representations*) reported poorer global QoL (*B* = −1.74), and poorer social functioning (*B* = −2.07). All significant associations were of trivial clinical importance according to the EORTC guidelines.^28^

**TABLE 3:** Regression analyses relating illness perceptions to health‐related quality of life while controlling for sociodemographic and clinical characteristics

Illness perceptions	Health‐related quality of life											
	Overall QoL		Physical functioning		Role functioning		Cognitive functioning		Social functioning		Emotional functioning	
	*B*	*P*	*B*	*P*	*B*	*P*	*B*	*P*	*B*	*P*	*B*	*P*
Consequences	**−1.95**	**<.01** **	**−1.97**	**<.01** **	**−3.03**	**<.01** **	−0.46	.57	**−2.31**	**<.01** **	−1.66	.02*
Timeline	0.28	.37	0.39	.18	0.44	.30	0.05	.91	0.37	.36	0.56	.10
Personal control	−0.14	.73	0.63	.09	0.08	.78	0.16	.75	0.35	.48	0.18	.67
Treatment control	**−1.67**	**<.01** **	**−1.28**	**<.01** **	−1.17	.04*	−1.13	.04	−1.39	.01**	0.29	.53
Identity	−1.09	.03*	−0.31	.51	−1.07	.12	−1.26	<.05*	**−1.83**	**<.01** **	−1.18	.03*
Concern	0.09	.88	−0.10	.85	−0.76	.33	0.59	.41	1.22	.10	−0.44	.49
Emotional representation	**−1.74**	**<.01** **	−0.16	.77	−0.17	.83	−1.14	.11	**−2.07**	**<.01** **	−1.36	.03*
Illness understanding	0.47	.30	−0.25	.56	0.56	.36	−0.48	.40	−0.14	.81	−0.48	.32

Illness perceptions	Health‐related quality of life																	
	Fatigue		Nausea		Pain		Dyspnoea		Insomnia		Appetite		Constipation		Diarrhea		Financial impact	
	*B*	*P*	*B*	*P*	*B*	*P*	*B*	*P*	*B*	*P*	*B*	*P*	*B*	*P*	*B*	*P*	*B*	*P*
Consequences	**3.13**	**<.01** **	0.70	.09	**2.59**	**<.01** **	1.56	.04*	1.94	.07	0.55	.41	0.78	.28	0.90	.15	0.44	.54
Timeline	−0.24	.53	−0.06	.74	−0.75	.07	−0.28	.44	−1.07	.04*	−0.40	.22	0.20	.57	0.25	.40	0.17	.63
Personal control	−0.10	.84	−0.03	.90	0.35	.48	−0.54	.22	0.39	.54	−0.77	.05	0.05	.91	−0.50	.18	0.70	.11
Treatment control	0.62	.24	0.27	.31	0.82	.14	0.62	.21	0.44	.53	0.29	.51	−0.20	.67	0.13	.76	0.60	.21
Identity	**2.15**	**<.01** **	0.27	.40	0.79	.23	1.26	.03*	**2.51**	**<.01** **	0.82	.12	**1.55**	**<.01** **	0.45	.35	**1.95**	**<.01** **
Concern	0.78	.29	0.52	.14	0.03	.97	0.25	.71	−0.01	.99	0.70	.23	−0.70	.28	−0.76	.17	−0.63	.33
Emotional representation	−0.21	.78	0.53	.13	0.68	.36	0.19	.77	0.96	.30	1.34	.02*	0.59	.35	0.95	.08	0.03	.96
Illness understanding	−0.26	.64	−0.03	.91	−1.33	.03*	0.46	.38	−1.00	.18	−0.38	.42	−0.12	.81	0.06	.89	−0.03	.96

*Note:* Regression analyses controlled for sociodemographic (sex, age, marital status, educational level, and work status) and clinical characteristics (time since diagnosis, type of DTC, cancer stage, primary treatment and number of co‐morbidities). All statistically significant values are given in bold. *B* = regression coefficient, *P* = *P* value for significance.

* *P* < .05

** *P* < .01.

Overall, in addition to the associations with illness perceptions, the following covariates were significantly related to HRQoL and of small clinical relevance. DTC survivors of older age reported poorer role functioning, whereas females reported less financial difficulties. Those who were not married or living together reported more problems of constipation. An increasing number of co‐morbidities was related to poorer global QoL, and physical functioning, and more pain. No significant relations were found for the remaining sociodemographic or clinical covariates.

## 4 DISCUSSION

The main finding of this study is that DTC survivors holding stronger beliefs their illness affects their live (*consequences*); their illness can be controlled by treatment (*treatment control*); they are emotionally affected by their illness (*emotional representation*) report a poorer HRQoL, independent of sociodemographic and clinical characteristics. Specifically, our study showed that DTC survivors who belief their life is negatively affected by their illness (*consequences*) report poorer global QoL, physical, role, and social functioning, whereas they report more fatigue, and pain. Furthermore, DTC survivors who belief their illness can be controlled by treatment (*treatment control*) report a poorer global QoL, and lower scores on physical functioning. Those DTC survivors who attribute many symptoms to their illness (*identity*) report more fatigue, insomnia, and a larger financial impact. DTC survivors who report to be more emotionally affected by their illness (*emotional representations*) report a poorer global QoL, and social functioning.

Although the majority of our findings are in line with previous studies^29^, ^30^, ^31^; we surprisingly found that contrary to studies among other cancer survivors, DTC survivors with a strong belief treatment could control their illness reported a poorer global QoL, and physical functioning. This negative association may be explained as followed. DTC survivors need life‐long thyroid replacement therapy after being treated with a total thyroidectomy. In preparation of follow‐up scans examining thyroid cancer recurrence, thyroid treatment is stopped. During this withdrawal period, hypothyroidism may occur which has known negative impacts on HRQoL.^32^ Moreover, even while on constant and adequate dosage of thyroid replacement therapy, many side effects negatively impacting HRQoL are known among non‐cancer populations.^33^ However, studies report that—while having clinically adequate blood levels—non‐cancer populations suffering from thyroid disease who had a total thyroidectomy have better HRQoL levels compared to those without previous surgery who solely receive hormone replacement therapy.^34^, ^35^ Noteworthy, health care professionals acknowledge and underscore the difficulty of attributing symptoms to thyroid disease when with hormones‐treated patient's clinical blood levels are within range.^36^ Extra caution is therefore warranted when administering patients' additional thyroid hormones, just in case symptom are from their thyroid disease.^36^

Interestingly, disease understanding was unrelated to HRQoL in our DTC survivors, although previous studies among other patient populations have also reported nonsignificant findings.^17^, ^29^ A former study among DTC patients revealed that a higher level of disease understanding was associated however with less negative emotional perceptions.^20^ This suggests that disease understanding maybe indirectly related to HRQoL through negative emotional perceptions. Indeed, in a study conducted by our own group we found that greater information support was associated with better illness perceptions, and that better illness perceptions were associated with less distress.^22^

The findings of our study showed that negative Illness perceptions are related to poorer HRQoL among DTC survivors. As a result, DTC survivors' HRQoL may be improved by changing these negative illness perceptions. A previous study by our group has shown that better information provision was associated with more positive illness perceptions among various cancer survivors.^22^ This relation was not limited to illness understanding but also related to cognitive and emotional perceptions.^37^ It is key that the provided information is tailored to the individual patient. However, as a previous study among gynecological cancer survivors has shown that providing extensive information in the form of a cancer survivorship plan can result in poorer illness perceptions.^38^

### 4.1 Clinical implications

Prior studies have suggested that sense of coherence (SOC) may be a pathway, mediating the relation between illness perceptions and HRQoL,^39^ as patients with a strong SOC define their illness as comprehensible, manageable and meaningful.^40^ Improving DTC survivors SOC can therefore help them with being less emotionally affected by their illness, developing more appropriate cognitions and acquiring a higher understanding of their illness, and therefore improving their HRQoL. Although interesting, research into the role of SOC within the context of HRQoL has exclusively focused on congenital heart disease, therefore further research would be needed to examine its effect among DTC survivors.

Psychological interventions such as cognitive‐behavioral therapy may also help DTC survivors with having less negative beliefs about the potential consequences of their illness and therefore reduce certain negative emotions (by improving emotional illness perceptions) and improve their cognitions. Indeed, a review among patients with cardiovascular disease shows that cognitive‐behavioral therapy together with education and counseling are beneficial in changing illness perceptions.^17^ Among cancer survivors, studies have shown comparable results. A study found that positive changes in illness perceptions improved emotional well‐being among breast cancer patients who took part in a psychosocial aftercare program.^18^ Similarly, a previous study found that changes in illness perceptions (especially by increasing the belief of control) was related to positive changes in psychological well‐being over time within a sample of esophageal cancer survivors.^19^ Accordingly, the aforementioned studies provide promising evidence for the effectiveness of interventions aimed at helping individuals acquiring more adaptive illness perceptions, thereby improving their HRQoL. However, there are currently no studies looking into the effectiveness of illness perception interventions among DTC survivors, therefore future research within this specific population is warranted. Reforming illness perceptions may not only be beneficial for DTC survivors' HRQoL, but may also stimulate adequate health‐behaviors such as medication adherence.^41^ This is an important aspect of after‐care among DTC survivors as most depend on life‐long thyroid replacement therapy.

### 4.2 Limitations and strengths

Results of this study should be interpreted while keeping in mind the following limitations. We included DTC survivors (2‐20 years after diagnosis), therefore we introduced survivorship bias to our sample. We know a poorer HRQoL is a predictor of survival,^42^ hence those DTC survivors with poorer HRQoL could be underrepresented as they might have deceased prior to the study. Furthermore, our results showed that thyroid cancer survivors of older age, being female and having co‐morbid conditions reported a poorer HRQoL, although of small clinical significance. Respondents vs nonrespondents however showed no statistical differences in sociodemographic or clinical characteristics, therefore the impact seems to be limited. The cross‐sectional study design prohibits us from making causal inferences. Although our analyses do indeed imply a significant yet trivial relation between variables, cause‐and‐effect cannot be determined, meaning that illness perceptions and HRQoL may have a reciprocal relationship. Furthermore, other factors that are not included in this secondary data analysis, such as Type D personality^43^ or psychological functioning^7^ are also related to HRQoL. Although inclusion of many other factors may improve the model explaining variation in HRQoL, knowledge on changeable determinants like illness perceptions are most valuable when it comes to improving patient‐outcomes like HRQoL. Our study also contains several strengths, as this is the first study relating illness perceptions to HRQoL among DTC survivors. Furthermore, our study has a high response rate (85%), large sample size (N = 284), and included sociodemographic and clinical characteristics as covariates in the analyses to correct for potential confounding. Moreover, we included survivors who were diagnosed 2 to 20 years ago, representing mid to long‐term survivors. The effect of survivorship bias seems limited as the majority of DTC patients have a good survival rate.

### 4.3 Conclusion

DTC survivors with negative emotional and cognitive perceptions about their illness report poorer HRQoL independent of sociodemographic and clinical characteristics. In detail, negative emotional and cognitive perceptions were associated with poorer global, emotional and social functioning, and more symptoms of fatigue, nausea and vomiting, pain, dyspnoea, insomnia, appetite loss, and constipation. Interestingly, disease understanding was not related to any of the HRQoL scales. The negative effects of DTC on HRQoL may be affected by poor illness perceptions. This knowledge is therefore helpful in identifying DTC survivors at risk of experiencing a poor HRQoL, and given the modifiable nature of illness perceptions, it provides a possibility for improving them by means of psychological interventions.

## CONFLICT OF INTEREST

The authors declare no conflicts of interest.

## ETHICS STATEMENT

This study was reviewed by the Institutional Review Board and was deemed nonhuman subjects research.

## INFORMED CONSENT

All participants provided informed consent.
